# Early Rehabilitation for Cerebellar Complications Following Left Atrial Myxoma Excision: A Stitch in Time Saves Nine

**DOI:** 10.7759/cureus.28773

**Published:** 2022-09-04

**Authors:** Moli Jain, Pallavi Harjpal, Vaishnavi Yadav, Rakesh K Kovela, Vishnu Vardhan

**Affiliations:** 1 Department of Cardiorespiratory Physiotherapy, Ravi Nair Physiotherapy College, Datta Meghe Institute of Medical Sciences, Wardha, IND; 2 Department of Neuro Physiotherapy, Ravi Nair Physiotherapy College, Datta Meghe Institute of Medical Sciences, Wardha, IND; 3 Department of Physiotherapy, Nitte Institute of Physiotherapy, NITTE (Deemed to be University), Deralakatte, IND

**Keywords:** cardiac tumours, physiotherapy, cerebellar complications, rehabilitation, left atrial myxoma

## Abstract

The most known of all primary cardiac tumors is myxoma, which is most usually detected in the left atrium. As there are no physical signs or symptoms, a diagnosis is rarely made purely based on clinical evidence. Our study aims to investigate the case of post-operative left atrial myxoma with cerebellar signs. A 50-year-old woman complained of dizziness and syncope, which caused her to collapse on the floor early in the morning. Myxoma in the left atrium and mitral valve regurgitation was discovered after prompt medical assistance. She was recommended for surgery to excise the left atrial myxoma and mitral valve repair. Post the surgery, she developed breathing difficulties and cerebellar signs for which she was referred for physiotherapy. She underwent two weeks of tailor-made inpatient rehabilitation. This case study intends to emphasize the importance of early diagnosis, treatment, and, most importantly, rehabilitation to return the patient to her functional state. A structured exercise regimen assists the patient while also reducing post-surgery problems. Timely monitoring and treatment are projected to improve outcomes in patients treated with a multidisciplinary approach.

## Introduction

Cardiac tumors are uncommon entities in medicine. They are far more common in women and occur between the third and sixth decades of life. Cardiac myxoma is the most frequent of all primary cardiac tumors. Myxomas are most typically found in the left atrium, where they develop from a stalk linked to the atrial septum. Approximately 75% of myxomas are found in the left atrium, 20% in the right atrium, and 8% in the ventricles [1]. Patients have at least one of the standard triad symptoms: 1) obstructive cardiac concerns; 2) embolic symptoms, and 3) constitutional or systemic health conditions. It shows that this benign tumor can induce a variety of clinical symptoms, including cardiovascular disease and infective, immunologic, and neurologic disorders. Because there are no identifiable physical indications or symptoms, diagnosis is rarely made solely on clinical grounds. Myxoma is usually sporadic, though there have been reports of familial or repeated occurrences [2]. Echocardiography is a powerful tool for determining the position, attachment, form, size, and mobility of an intracardiac mass, as well as the existence and extent of any resulting hemodynamic disturbance [3].

Unusual appearances of cardiac myxomas provide diagnostic and differential diagnosis issues for physicians and can result in a delayed or missed diagnosis, inappropriate monitoring, delayed treatment, and possibly a bad prognosis. Greater knowledge of the atypical nature of cardiac myxomas will aid correct care and enhance prognosis. Because of the potential for malignancy, all patients without contraindications should have their tumours surgically resected as soon as possible after a diagnosis is made. The brain, lungs, bones, and soft tissues have all shown distant cardiac myxoma metastases before the diagnosis or several years after the surgical excision of the initial cardiac myxoma [4,5]. As in our instance, an uncommon appearance of cerebellum involvement can readily mask myxoma as the etiology of stroke as a differential diagnosis. For early diagnosis and prevention of problems, a high level of suspicion is required [6].

## Case presentation

A 50-year-old woman who worked as a farmer presented to the emergency room with the chief complaints of abrupt dizziness and syncope, which caused her to collapse to the ground in the early morning. She was immediately taken to the local hospital where emergency treatment was given after which various lab investigations were done. Echocardiography revealed myxoma in the left atrium and mitral valve regurgitation. She has advised emergency surgery for excision of left atrial myxoma and mitral valve repair. She was then referred to our hospital and admitted to the cardiovascular and thoracic surgery (CVTS) ICU for surgery. On postoperative day one, she was referred for physiotherapy. On postoperative day three, she had the following complaints: pain at the incision site, difficulty in breathing, difficulty maintaining balance, and difficulty performing the basic activity of daily living (washing, toileting, eating).

Clinical findings

On examination, the patient was medically stable, cooperative, oriented to person, place, time, and situation, and willing to participate in inpatient rehabilitation. She was medically alert, hemodynamically healthy, and competent upon review. With a blood pressure of 118/70 mm Hg, her vitals were stable. Pulmonary, cardiovascular, gastrointestinal, and thyroid tests were otherwise unremarkable. A neurological analysis showed jerky nystagmus to the left with a rapid motion. On both the upper and lower limbs, hypotonia (grade 1+, according to the tone rating scale) was present bilaterally. Sensory examination revealed impairment of proprioception, kinaesthesia, vibration, and two-point discrimination on both sides. All the non-equilibrium tests were grade two (moderate impairment: able to accomplish the activity, movements are slow, awkward, and unsteady) bilaterally. Balance test in sitting was graded fair, while in standing were poor. Romberg’s test was poor and tandem walking was absent. Planter reflex was mute bilaterally. Functional range of motion was present on both sides with medical research council grade three. On her left hand, past pointing was pronounced with heel shin testing affected on both sides. On gait assessment, the patient was able to walk on postoperative day 10, that too with minimal assistance from the caregiver. The gait deviations found were hip abduction (inability to shorten leg for limb clearance), limited knee flexion, trunk deviation towards the left, and decreased arm swing. She showed a wide-based gait and dysdiadochokinesia, as well as normal speech, all of which pointed to cerebellar involvement.

The complete sequence of events from the day of admission is shown in Table 1.

**Table 1:** Timeline of events TDS: *ter die sumendum* (three times a day); BD: *bis in die* (twice a day); OD: *omne in die* (once a day); CT: computed tomography; FiO2: fraction of inspired oxygen

S. No.	Date of Events	Consultation	Findings	Suggestions
1	March 28, 2022	Emergency	Dizziness and syncope, large fragile mass present over the chest. Echocardiography revealed myxoma in the left atrium and mitral valve regurgitation.	Inj. heparin- 5000 units/ml TDS, Tab. Cardivas 125 mg BD
2	April 4, 2022	Surgery	Atrial myxoma excision with mitral valve repair, patient on the mechanical ventilator (positive end-expiratory pressure-5 cm H_2_O, FiO_2_-50%). CT Brain revealed encephalomalacic changes in the cerebellar hemisphere.	Inj. ceftriaxone 1 mg BD, Inj. amikacin 500 mg OD, Inj. paracetamol 100 ml, Inj. ondansetron 4 mg, Inj. tramadol 50 mg, Inj. pantoprazole 40mg OD
3	April 5, 2022	Physiotherapist	Pain near the incision, breathing difficulties, and trouble keeping one's balance and coordination.	Nebulization, chest physiotherapy and suctioning, ankle foot toe movement, rolling facilitation, and transition training
4	April 18, 2022	Discharge	Improvement in balance and coordination.	Coordination training, strengthening exercises, gait training, and fine motor training, patient was discharged with a proper home exercise program.
5	May 2, 2022	Follow-up	The patient came walking by herself and there was an improvement in her gait pattern.	Strengthening exercises, gait training, and fine motor training continued.

Diagnostic assessment

Preoperative

**Figure 1:**
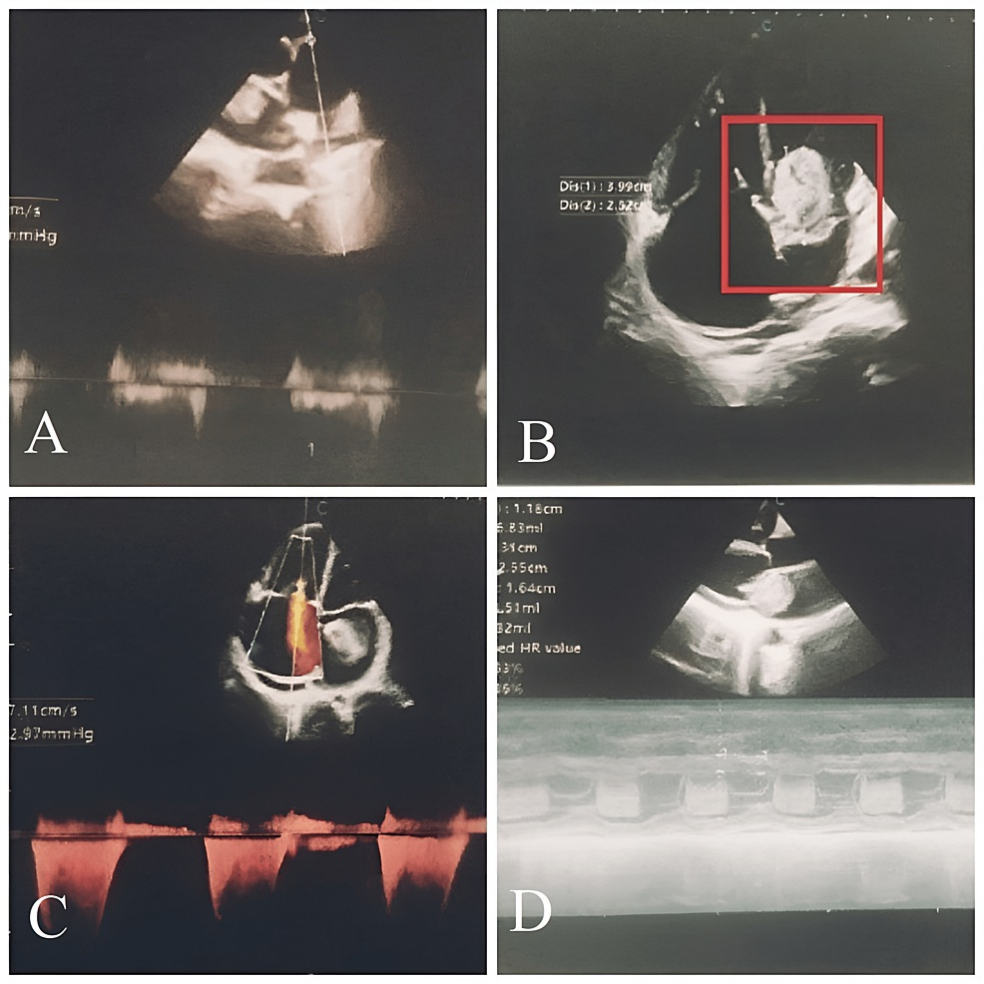
Preoperative echocardiography (A) dilated left atrium; (B) large left atrial mass measuring 3.99 X 2.52 cm, attached in the mid part of the interatrial septum; (C) mild left ventricle inflow obstruction; (D) left atrial mass encroaching left ventricle during systole and causing prolapse of the anterior mitral leaflet

On echocardiography, there was a short PR interval along with a complete right bundle branch block. Preoperative echocardiography (Figure 1) revealed a dilated left atrium, a large left atrial mass measuring 3.99 X 2.52 cm attached in the mid part of the interatrial septum, encroaching left ventricle during systole, and causing prolapse of the anterior mitral leaflet with mild left ventricle inflow obstruction. 

Postoperative

**Figure 2:**
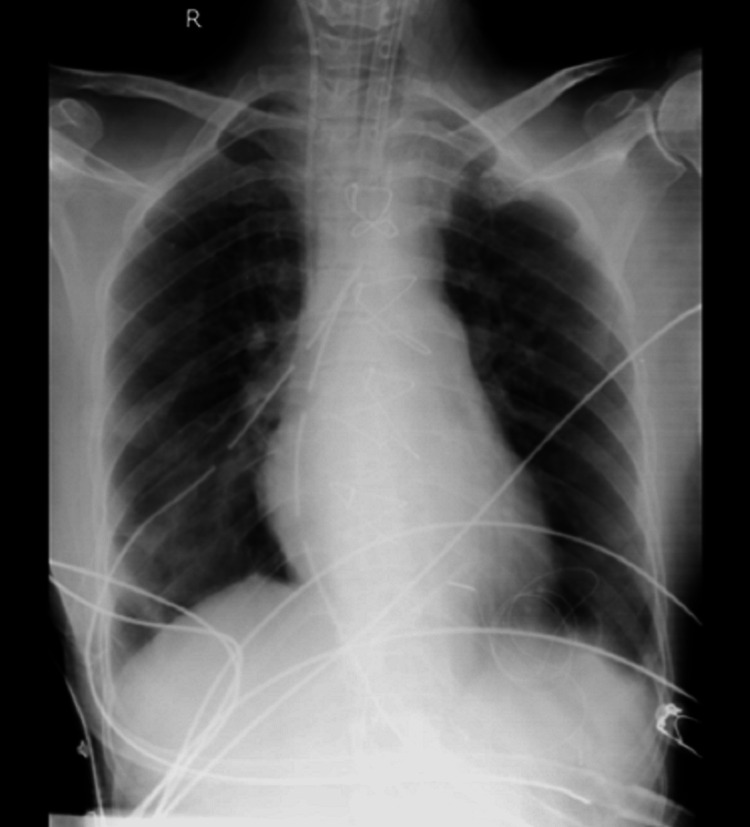
Postoperative chest x-ray PA view PA: posterior-anterior

**Figure 3:**
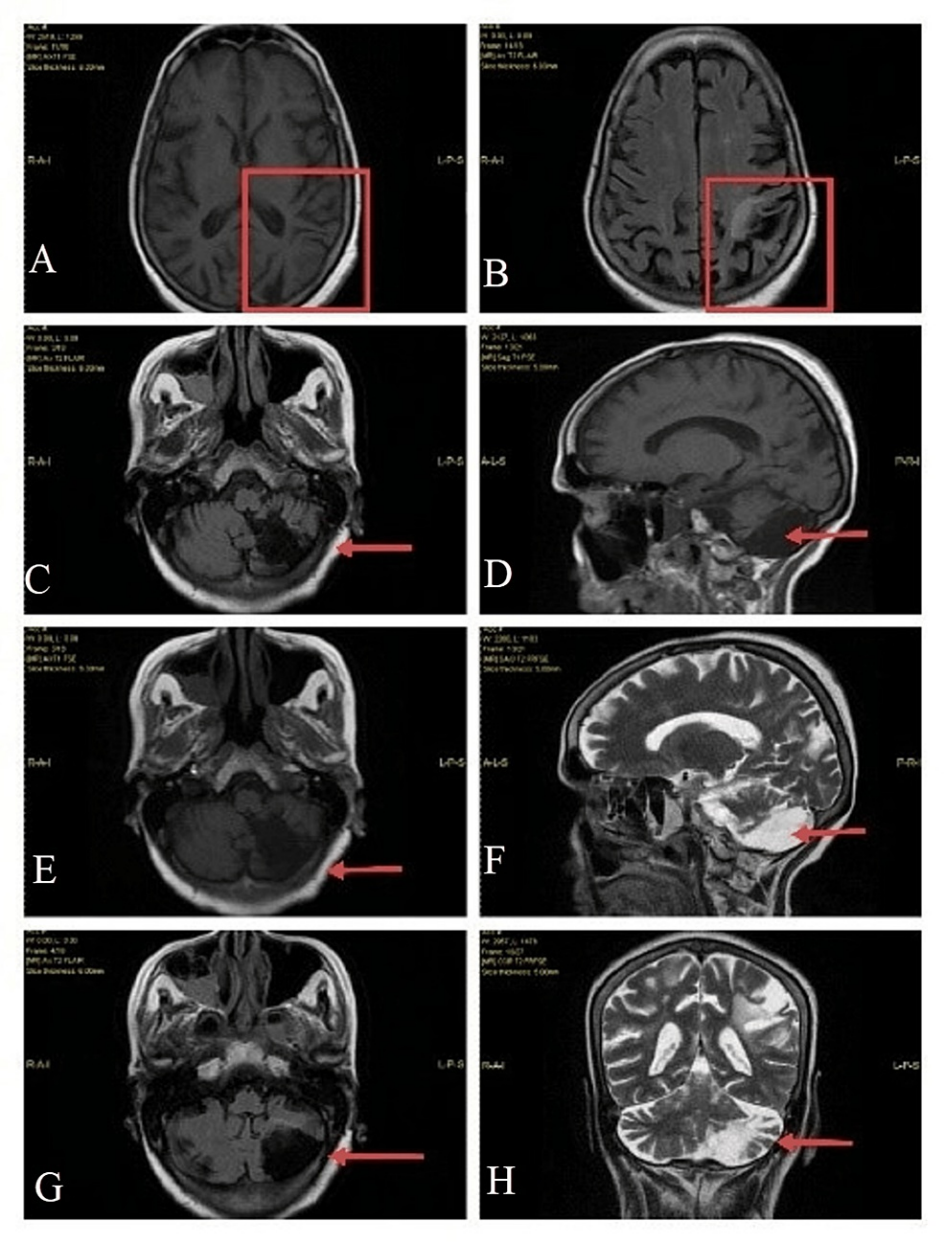
Postoperative MRI findings (A,B) gliotic changes in the left posterior parietal and occipital region; (C) gliotic changes (red arrow) in the left posterior parietal region; (D) encephalomalacia changes (red arrow) in the left cerebellar region; (E) gliotic changes (red arrow) in the left posterior parietal region; (F) encephalomalacia changes (red arrow) in the left cerebellar region; (H) encephalomalacia changes (red arrow) in the left cerebellar region; (G) gliotic changes (red arrow) in the left occipital region

Chest x-ray posterior-anterior (PA) view (Figure 2) showed right mid zone and left basal lung subtle patchy haziness. On MRI (Figure 3), there were gliotic changes in the left posterior parietal and occipital region, encephalomalacia changes in the left cerebellar region, and age-related cerebral atrophic changes with small vessel ischemic changes, and sinusitis.

Therapeutic interventions

**Figure 4:**
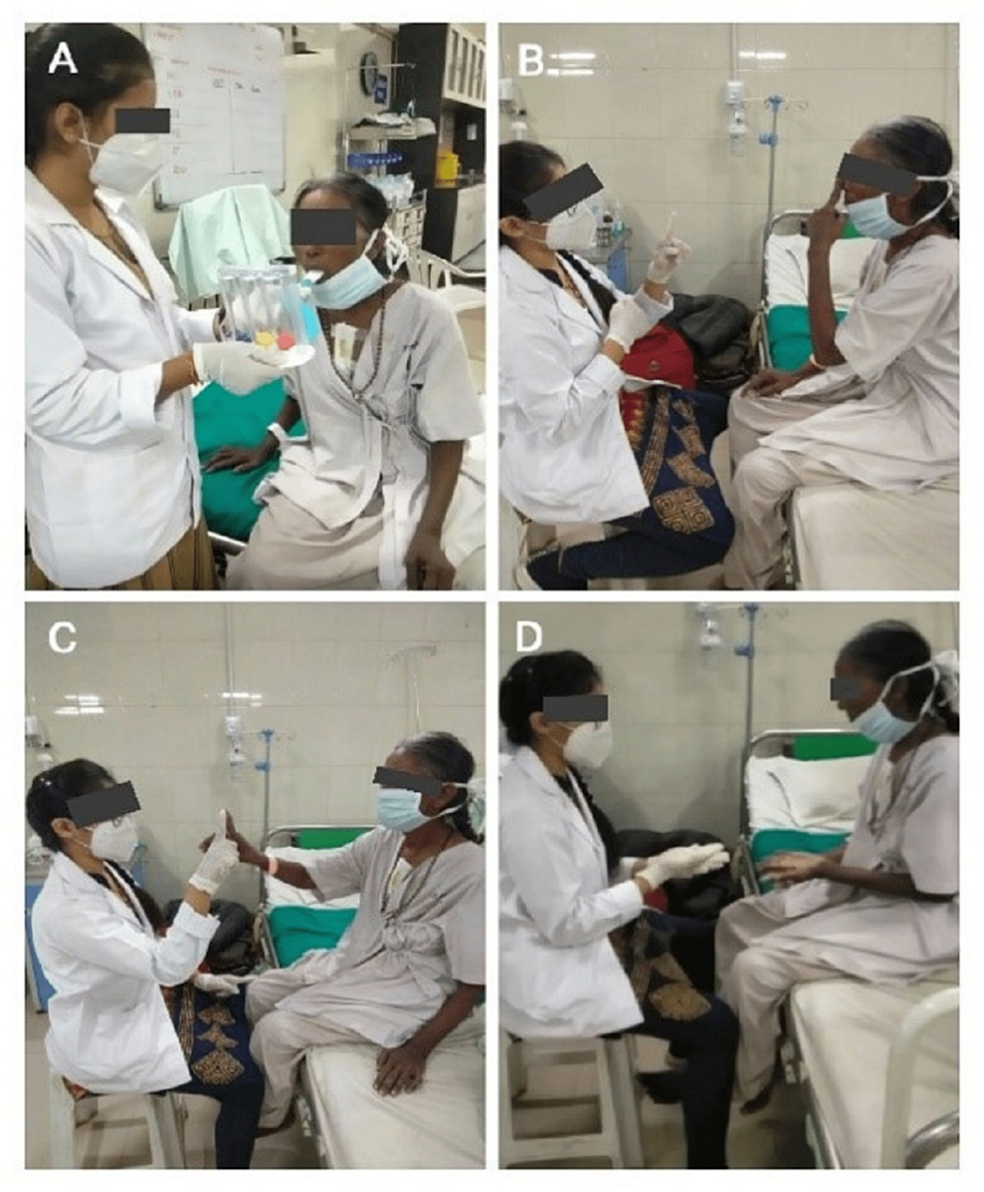
Demonstrating the therapeutic interventions (A) patient performing incentive spirometry; (B) coordination exercise: finger to nose; (C) coordination exercise: finger to therapist's finger; (D) coordination exercise: pronation/supination

The therapeutic intervention focused on early rehabilitation of the patient enabling her to perform activities of daily living. The patient was visited by physiotherapists twice daily for two weeks. Each rehabilitation session was for about 30-45 minutes with a proper rest period in between the exercises. The therapeutic interventions provided to the patient are depicted in Table 2 and Figure 4.

**Table 2:** Therapeutic Intervention provided to the patient

Problem identified	Probable cause	Goal Framed	Physiotherapy Intervention
Mild swelling in lower limbs	Reduced venous return and decreased mobility	To prevent deep vein thrombosis	Limb elevation (20-30 degrees three-four times/day for 15 minutes), ankle foot toe movement (start from 10 repetitions progressed to 30 repetitions at the end of the first week) along with calf strengthening.
Decreased air entry in lungs	Weakness of diaphragm and intercostal muscles	Improve the aeration of lungs with active contraction of the diaphragm	Diaphragmatic breathing, thoracic expansion exercises, and regular Incentive Spirometry (Figure 4A).
Accumulation of secretions	Decreased mobility and under the effect of anesthesia	To maintain bronchial hygiene	Nebulization, chest physiotherapy. (percussion and vibration in modified postural drainage position) followed by suctioning.
Inappropriate posture	Bedridden for many days postoperatively	To prevent postural defect	Chest binders and positioning every two hours.
Reduced bed mobility	A reduction in pulmonary and muscular endurance as well as weakness	Improve bed mobility and prevent pressure sores	Transition training and rolling facilitation were promoted even after the ICU was moved to the ward.
Decreased out-of-bed transitions	Weakness in girdle muscles and decreased stability	Increase functional performance	Training for transitions from lying down to sitting and standing up.
Impaired proprioception	Cerebellar involvement	Improve proprioception	Proprioceptive training and joint compression.
Impaired coordination	Cerebellar involvement	Improve coordination	Finger to the nose (Figure 4 [B]), finger to the therapist's finger (Figure 4 [C]), alternate nose to finger, finger opposition, pronation/supination (Figure 4 [D]), heel to the shin, and Frankel exercises are some examples of coordination training.
Reduced sitting balance	Cerebellar involvement and prolong hospital stay	Improve Sitting balance	Sitting balance training like proprioceptive neuromuscular facilitation taught – alternating isometrics and rhythmic stabilization along with perturbations in a safe manner with a variety of surfaces.
Reduced strength	Weakness and hospital stay	To improve strength	Upper limb strengthening with a water bottle (half a liter initially progressed to one liter) lower limb strengthening with weight cuff (half kg initially progressed to one kg) hip hikers strengthening along with quadriceps strengthening.
Impaired walking pattern	Prolong hospital stay and cerebellar improvement	Gait training	Seated marching, knee extension, toe taps, knee to chest, single leg stance, side leg raises, ankle dorsiflexion, toe raises, and heel raises.
Decreased activities of daily living	Decreased performance of muscles	Advise the patient to be as active as possible	Encouraged how to use the extremities to involve in activities of daily living.

Follow-up and outcome of interventions

The outcome measures before rehabilitation and on discharge are shown in Table 3. There was a tremendous improvement in the patient and the outcome measures post-rehabilitation.

**Table 3:** Follow-up and outcome of interventions POD: postoperative day

Outcome Measure	Pre-Rehabilitation	Discharge
Visual Analogue Scale (POD 4)	On rest: 4; On activity: 7	On rest: 0; On activity: 2
Borg rate of perceived exertion (POD 4)	Grade-3, moderate	Grade 0.5, very, very slight (just noticeable)
Incentive spirometry (POD 4)	900 cc	1200 cc
Berg balance scale (POD 7)	Score 6/56	Score 33/56
Two-minute walk distance test (POD 10)	Distance covered: 42 m	Distance covered: 102 m

## Discussion

This case report was on a 50-year-old woman who underwent surgery to remove a left atrial myxoma and repair her mitral valve. Following surgery, she underwent two weeks of an in-hospital cardiac rehabilitation program that was specifically designed for her. At the two-week follow-up after discharge, she showed improvements in her gait and quality of life. From a rehabilitation point of view, this case is rare and unique; firstly, because of the myxoma of the size of 3.99 X 2.52 cm along with mitral valve prolapse, secondly, its postoperative presentation with cerebellar involvement, and lastly, the challenging multidisciplinary post-rehabilitation and positive outcomes.

In addition, the diverse and atypical clinical manifestations of a cardiac myxoma vary from case to case. The main cause of the poor prognosis is the delayed onset of the symptoms and delay in the diagnosis. A thorough understanding, early diagnosis, and treatment of cardiac myxoma are of utmost importance.

In designing the protocol, we used the previous shreds of evidence that state that the initiation of early cardiac rehabilitation improves one's quality of life while lowering medical expenses [7] and shows marked improvement in exercise performance, six-minute walk test, and quality of life after the cardiac surgery [8]. For the neurological complications in our patient, the rehabilitation program was designed in line with the study that suggests postural control, balance and coordination training, and gait training, with or without the use of orthotic devices and aids for ataxic gait following cerebellar lesions [9]. Similarly, a systematic review stated balance exercises, coordination training, aerobic exercises like cycling, gait training, range of motion, and strengthening exercises for the extremities or a combination of these exercises for such patients [10]. These exercises also facilitate motor performance and diminish ataxia symptoms, enabling patients to accomplish personally significant daily goals [11].

## Conclusions

This case demonstrates the importance and utility of early inpatient rehabilitation following surgery. The multidisciplinary rehabilitation program designed for the patient will add to the existing literature on physical therapy among such patients. To get the patient back to her functional state, early diagnosis, treatment, and most importantly, rehabilitation played important roles. A regular exercise regimen helps the patient and reduces postoperative problems. In patients treated with a multidisciplinary approach, timely monitoring and treatment are expected to improve patient outcomes. 
